# Anatomy of the Ovum, in the Ovary

**Published:** 1836-04

**Authors:** Thomas Wharton Jones


### ANATOMY OF THE OVUM, IN THE OVARY. BY THOS. WHARTON JONES, ESQ.

[THE following is a brief abstract of a paper read before the Royal Society, 18th June, 1835. Although preceded by M. Coste, whose memoir was reported on to the Royal Academy of Sciences, 5th May, 1834, we have reason to believe the enquiries of Mr. Jones to be perfectly original, and regret they have not appeared in the second part of the Philosophical Transactions for 1835, just published.]

§ 1. The human ovary, like that of the mammalia generally, consists of a parenchymatous substance, enclosed within a fibrous tunic called *tunica albuginea,* and covered by peritoneum. The vesicles of De Graaf are imbedded in the parenchyma, and in the human species measure, when mature, about one-fifth of an inch in diameter.

§ 2. The proper capsule, or *theca* of the Graafian vesicle, is composed of two layers; the *outer* of which is thin, dense, and vascular; the *inner* thicker, softer, and more opaque. It is the latter which, after impregnation, forms the *corpus luteum.*

§ 3. The contents of the Graafian vesicle consist of the following parts:

1st. A granular* membrane.

2d. Inclosed within the granular membrane, a coagulable granular fluid, forming by far the greater part of the contents of the vesicle.

* By the term *granular,* applied to a membrane, is meant that, under the microscope, it breaks up into granules, previously kept together by a mucus-like substance.

3d. Connected with the granular membrane, and sustained by the fluid just mentioned, a circular mass or disc, termed by Baer the *proligerous** *disc*, which, at its centre and on its exterior surface, is hollowed out into a cuplike cavity.

4th. The *ovum*, properly so called, held within the cavity of the proligerous disc.

§ 4. The ovum, as it existed in the ovary, was first observed, in the bitch, by Professor Baer, of Koningsberg, in 1827. The human ovum is so small as to be only just perceptible to the naked eye, being $\frac{1}{150}$th of an inch in diameter: it possesses a soft transparent capsule of considerable thickness, lined by a granular membrane, the granules of which adhere together by the intervention of a delicate mucus-like substance, and contains, besides the vesicle of Purkinje, presently to be noticed, an albuminous-like fluid.

§ 5. Imbedded in the granular membrane of the human ovum, Mr. T. Wharton Jones has discovered† with the microscope a delicate transparent vesicle, about $\frac{1}{900}$th of an inch in diameter. The same vesicle has been seen in the ovum of the rabbit, by M. Coste. Mr. Jones describes it as having on one side a small elevation, which, projecting among the granules of the granular membrane of the ovum, fixes it in its place. He considers this vesicle as analogous to that described by Professor Purkinje‡ in the cicatricula of the immature eggs of birds, and which exists also in the ova of other oviparous animals. Mr. Jones has examined the ova of the cow, sheep, sow, rabbit, rat, and mouse, and has found in all these animals a similar vesicle.

§ 6. At impregnation, the granular membrane and capsule of the Graafian vesicle burst, as well as the tunica albuginea and peritoneal covering of the ovary, and the ovum (§ 4) escapes into the Fallopian tube. After this event, the vesicle discovered by Mr. Jones and Mr. Coste is no longer to be found in the ovum; a fact analogous to the observation of Purkinje, that the vesicle described by him has completely disappeared by the time when the bird's egg enters the oviduct.

§ 7. Although there is, at first sight, a considerable resemblance between the contents of the Graafian vesicle and the immature yelk of the egg of a bird, Mr. Jones thinks, contrary to the opinion of Baer, that there is no real analogy between them; because, in the Graafian vesicle of the mammalia, there is no membrane surrounding its contents, similar to the vitallary membrane of the ovum in birds; nor does the latter membrane appear first under the form of a granular membrane. Neither can the vesicle of Purkinje be considered the analogue of the ovum. The vesicle of Purkinje consists merely of a delicate capsule containing a fluid; while, in the ovum of the mammalia, there are found all the essential elements of the egg of birds and other ovipara, namely, an external membrane, analogous to the vitallary membrane, but performing a different function; a granular membrane, containing a thin fluid, corresponding to the immature yelk of a bird's egg; and a vesicle in every respect analogous to that which Purkinje found in the hen's egg, while still lodged in the ovary. Mr. Jones considers the granular membrane, proligerous disc, and granular fluid of the Graafian vesicle, as parts which are superadded, and of which there is no trace within the capsule of the ovary of a bird.

---

### OBITUARY.

### SIR DAVID BARRY, M.D.

In our last number we announced the sudden and premature death of this amiable and estimable physician: we now add a few particulars of his life, omitted for want of room.

Sir David Barry was born on the 12th March, 1780, in the county of Ros

* *Proligerous*, from *proles*, progeny, and *gero*, carry.

† Récherches sur la Génération des Mammifères. Paris, 1834.

‡ Symbolée ad Ovi Avium Historiam ante Incubationem. Lipsiæ, 1830.—Purkinje first described the vesicle which bears his name in 1825.

